# Mental Health Protection Programme BOJE (Colours) for LGBTQIA+ people in Croatia – Empowering Resilience

**DOI:** 10.1192/j.eurpsy.2025.870

**Published:** 2025-08-26

**Authors:** J. Hubler, A. Vuk, I. Barun, L. Mijalkovic, M. Supe, J. Pravdic, V. Grosic

**Affiliations:** 1 Substance use and Addiction Treatment Day care unit; 2 Department of integrative psychiatry; 3 Department of psychiatry, University Psychiatric Hospital Sveti Ivan, Zagreb, Croatia

## Abstract

**Introduction:**

The phenomenon of minority stress frequently emerges as a contributing factor to mental health discrepancies among sexual and gender minority people, manifesting in elevated rates of mental distress, anxiety, depression, suicidality and substance misuse (Plöderl et al. Int. Rev. Psychiatry 2015; 27 367-385, Russell et al. Annu Rev Clin Psychol. 2016; 12 465–487). Building on insights from previous research, the Mental Health Protection Programme BOJE (Colours) was launched to offer comprehensive support tailored to the needs of LGBTQIA+ individuals within the Croatian public mental healthcare system.

**Objectives:**

The presentation of the BOJE - Mental Health Protection Programme that aims to address minority stress to offer specialized support tailored to the unique needs of LGBTIQ+ individuals, fostering resilience and well-being.

**Methods:**

In October 2023, a multidisciplinary team at the University Psychiatric Hospital Sveti Ivan in Zagreb, Croatia, formed a Mental Health Protection Programme BOJE which consists of a counseling center, an outpatient clinic and a three-month therapeutic and educational cycle for LGBTQIA+ users. Additionally, a platform was provided for training healthcare professionals in LGBTQIA+ affirmative practice. The goal is to offer support, create an inclusive, safe environment and raise awareness of LGBTQIA+ mental health needs.

**Results:**

To date, 50 participants have been supported, with 50% identifying as TGD. We had approximately 574 procedures (counsellings, psychiatrist consultations and reviews and psychotherapies) and two cycles of closed-group workshops have been completed with a low dropout rate, and most participants rated the program as useful or very useful for their mental well-being.

**Conclusions:**

Despite the recognition of the mental health disparities between sexual and gender minority people and the general population there is still limited availability of gender-affirming mental and physical health services. The investigation of how affirming healthcare access influences resilience building among transgender and gender non-binary individuals highlighted the urgent need for additional interventions in public healthcare systems. Following this, a mental health program for LGBTQIA+ individuals was launched within the Croatian healthcare system to buffer the negative health effects of minority stress and improve mental health among sexual minorities and gender-diverse populations.

**Disclosure of Interest:**

None Declared

